# A nickel iron diselenide-derived efficient oxygen-evolution catalyst

**DOI:** 10.1038/ncomms12324

**Published:** 2016-08-09

**Authors:** Xiang Xu, Fang Song, Xile Hu

**Affiliations:** 1 Laboratory of Inorganic Synthesis and Catalysis, Institute of Chemical Sciences and Engineering, Ecole Polytechnique Fédérale de Lausanne (EPFL), EPFL-ISIC-LSCI, BCH 3305, Lausanne CH 1015, Switzerland

## Abstract

Efficient oxygen-evolution reaction catalysts are required for the cost-effective generation of solar fuels. Metal selenides have been reported as promising oxygen-evolution catalysts; however, their active forms are yet to be elucidated. Here we show that a representative selenide catalyst, nickel selenide, is entirely converted into nickel hydroxide under oxygen-evolution conditions. This result indicates that metal selenides are unstable during oxygen evolution, and the *in situ* generated metal oxides are responsible for their activity. This knowledge inspired us to synthesize nanostructured nickel iron diselenide, a hitherto unknown metal selenide, and to use it as a templating precursor to a highly active nickel iron oxide catalyst. This selenide-derived oxide catalyses oxygen evolution with an overpotential of only 195 mV for 10 mA cm^−2^. Our work underscores the importance of identifying the active species of oxygen-evolution catalysts, and demonstrates how such knowledge can be applied to develop better catalysts.

## Abstract (web summary)

Metal selenides have been reported as promising oxygen-evolution catalysts but their active forms are yet to be elucidated. Here, the authors show that metal selenides are unstable under oxygen-evolution conditions and that *in situ* generated metal oxides/hydroxides are responsible for their high activity.

## Graphical abstract

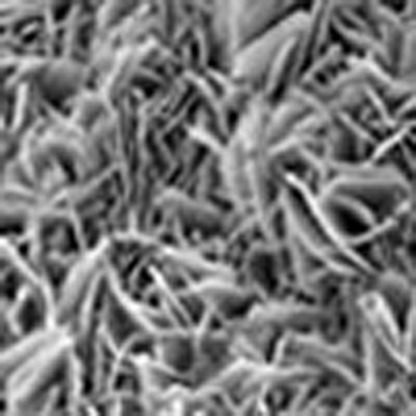

Sunlight-driven water splitting or carbon dioxide (CO_2_) reduction to make solar fuels is a promising solution to solar energy storage^1^. Essential to the water splitting and CO_2_ reduction reactions is the oxygen-evolution reaction (OER). This reaction is kinetically sluggish and demands an efficient electrocatalyst^2^. Although noble metal-based catalysts such as IrO_2_ and RuO_2_ exhibit good OER activity, their scarcity and high cost pose great constrains for large-scale applications. Tremendous efforts have been made in recent years to develop non-precious OER catalysts^3^,^4^. Despite the progress, a significant overpotential is still required for state-of-the-art catalysts. To reach 10 mA cm^−2^, a widely used figure of merit equivalent to ∼12% solar to hydrogen efficiency, nearly all non-precious catalysts need an overpotential of more than 250 mV.

The majority of non-precious OER catalysts are metal oxides and (oxy)hydroxides^3^,^4^. Recently, a few non-oxide-based OER catalysts including metal phosphides, sulfides and selenides are reported^5^,^6^,^7^,^8^,^9^,^10^,^11^. Given the limited stability of these compounds under highly oxidative potentials in alkaline solutions, questions have arisen on the nature of the true active species. Indeed, we and others showed that the surfaces of Ni_2_P and CoP were transformed into metal oxides during catalysis, which were responsible for the catalytic activity^5^,^6^,^7^. For Ni, Co and Fe sulfides, Chen *et al*. showed that they were entirely transformed into the corresponding metal oxides during OER^8^. Since metal selenides have similar chemical reactivity to metal sulfides, we were surprised by previous reports which suggested stability of bulk NiSe, Ni_3_Se_2_ and CoSe_2_ materials under OER^9^,^10^,^11^. Using a post-catalytic analysis, we show here that NiSe is completely converted into nickel hydroxides during OER, indicating that metal oxides or hydroxides are the active and final forms of metal selenides pre-catalysts in OER. This knowledge promoted us to purposely use metal selenides as templating precursors to highly active metal oxide OER catalysts, because methods to produce ultrasmall nanostructured metal selenides are readily available^12^,^13^,^14^,^15^. Following this strategy, we synthesize a hitherto unknown selenide, nickel iron diselenide (Ni_*x*_Fe_1−*x*_Se_2_), which upon *in situ* transformation into oxides, catalyses OER with an overpotential of only 195 mV for a current density of 10 mA cm^−2^. This is until now the most active single-phase OER catalyst in alkaline solutions. The high activity of this Ni_*x*_Fe_1−*x*_Se_2_-derived catalyst is largely due to its desirable nanostructure, inherited from its selenide precursor.

## Results

### Active form of NiSe in OER

**Figure 1:**
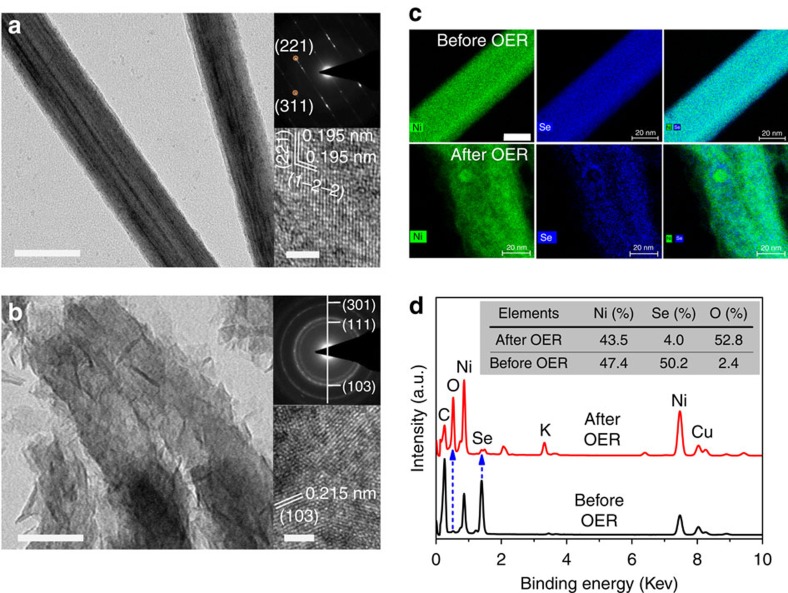
Structural and compositional characterization of NiSe. (**a**,**b**) TEM and HRTEM images and SAED patterns before (**a**) and after (**b**) OER. (**c**,**d**) Elemental mapping images and corresponding EDS before and after OER. Colours in elemental mapping images: green for Ni; and blue for Se. Cu and C signals in the EDS are from the copper grid and carbon film that are used to support the sample for TEM measurements. *K* is due to the residual electrolyte (1 M KOH). The inset in the spectra shows the elemental atomic percentages. Scale bar: (**a**) 50 nm; and (**b**) 50 nm. Insets in **a**,**b**, 2 nm; and **c**, 20 nm. EDS, energy-dispersive spectroscopy.

NiSe was synthesized via a hydrothermal approach using Ni foam as the precursor^9^. The electrocatalytic activity of NiSe towards OER was investigated in 1 M KOH using a three-electrode electrochemical system. Galvanostatic scan at the current density of 10 mA cm^−2^ was used to activate the catalyst. The activity was then measured by linear sweep voltammetry (LSV) at a scan rate of 1 mV s^−1^. The overpotentials to reach 10 mA cm^−2^ was ∼253 mV (Supplementary Fig. 1), in agreement with previous reports. The morphology and composition of the catalyst after catalysis for 12 h were then examined. Transmission electron microscopy (TEM) image showed that single-crystal nanowires of NiSe were converted to polycrystalline particles made of ultrathin nanosheets (Fig. 1a,b). Selected area electron diffraction (SAED) pattern of the sample after OER can be indexed to (111), (103) and (301) planes of α-Ni(OH)_2_ (Fig. 1b) (space group: *P*-31m, JCPDS No. 22-0444). The different lattice fringes in high-resolution TEM (HRTEM) images of samples before and after OER confirmed the total conversion of NiSe into Ni(OH)_2_. Similarly, the elemental mapping of the sample after OER showed that the Se content decreased from 50.2% to 4.0%, but the oxygen content increased from 2.4 to 52.8% during the transformation (Fig. 1c,d). Thus, Se was nearly completely removed while oxygen was incorporated during OER. The above data indicate that NiSe is entirely converted into Ni(OH)_2_ under OER conditions, which is the active form of the catalyst. Interestingly, the activity of the NiSe-derived Ni(OH)_2_ is higher than the most active Ni(OH)_2_ nanoparticles prepared by direct synthesis, which requires 300 mV to reach 10 mA cm^−2^ (refs 16, 17). This result suggests that metal selenides may serve as templating precursors to metal oxides or hydroxides with superior OER activity than those prepared by other methods. With this in mind, we turn our attention to nickel iron selenides, as NiFeO_*x*_ is one of the most active OER catalysts^18^,^19^,^20^,^21^.

### Fabrication of Ni_*x*_Fe_1−*x*_Se_2_

Metal selenides normally exist in two forms, mono-selenide (MSe) and diselenides (MSe_2_). We attempted to synthesize both Ni_*x*_Fe_1−*x*_Se and Ni_*x*_Fe_1−*x*_Se_2_. However, only diselenide Ni_*x*_Fe_1−*x*_Se_2_ could be obtained. NiSe_2_ and FeSe_2_ are well-known substances, while Ni_*x*_Fe_1−*x*_Se_2_ is a hitherto unknown selenide, probably due to a mismatch between the crystal structures of NiSe_2_ and FeSe_2_. Both have a cubic structure (space group: Pa-3), but NiSe_2_ has a cell parameter of *a*=5.960 Å, while FeSe_2_ has a parameter of *a*=5.776 Å. To circumvent this mismatch, a reductive solvothermal process was applied using NiFe-layered double hydroxide (LDH) grown on Ni foam as the precursor. We hypothesized that Ni and Fe were already well dispersed in the crystal lattices of NiFe LDH, so that its selenization might lead to pure-phase Ni_*x*_Fe_1−*x*_Se_2_ without phase separation between NiSe_2_ and FeSe_2_. This approach indeed worked.

**Figure 2:**
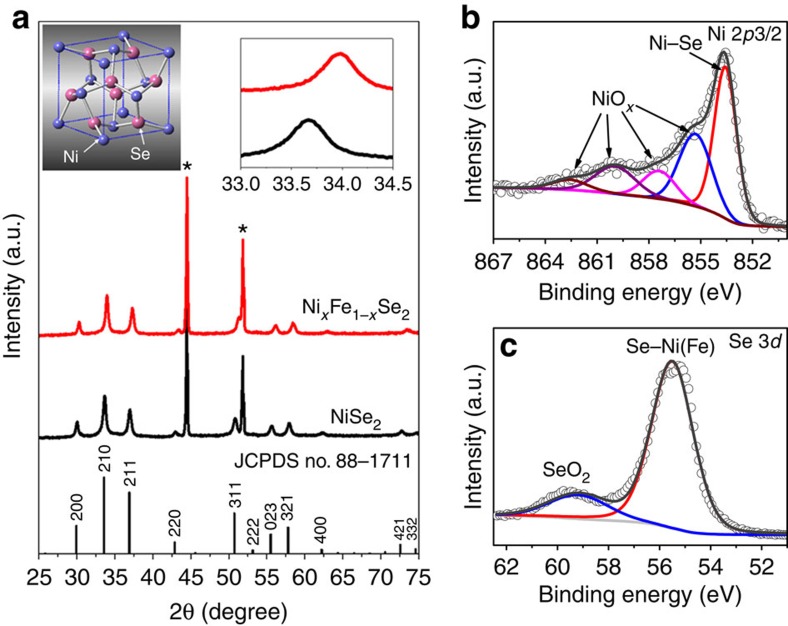
Characterization of Ni_*x*_Fe_1−*x*_Se_2_. (**a**) PXRD patterns. The asterisks ‘*' mark the diffraction peaks from the nickel foam substrate. The insets show the unit cell of cubic metal diselenides and the magnified PXRD patterns in the range between 33 and 34.5°. (**b**,**c**) High-resolution XPS of Ni 2*p*3/2 (**b**) and Se 3*d* (**c**). The main peaks (red) are attributed to Ni_*x*_Fe_1−*x*_Se_2_, while the minor peaks are assigned to surface impurities of metal oxides and SeO_2_.

Powder X-ray diffraction (PXRD) pattern (Fig. 2a) of the as-synthesized Ni_*x*_Fe_1−*x*_Se_2_ (*x*=0.8) indicated the formation of a cubic pyrite-phase metal selenide, similar to NiSe_2_ (space group: *Pa*-3, JCPDS No. 88–1711). The unit cell of cubic metal diselenides is shown in the inset in Fig. 2a. No crystalline impurity was detected. The diffraction peaks of Ni_*x*_Fe_1−*x*_Se_2_ were shifted to higher angles compared with their counter parts in NiSe_2_ (upper right inset in Fig. 2a). The crystal parameter of Ni_*x*_Fe_1−*x*_Se_2_ is *a*=5.884 Å, which is between NiSe_2_ (*a*=5.960 Å) and FeSe_2_ (5.776 Å). Ni_*x*_Fe_1−*x*_Se_2_ samples with other Ni:Fe rations (*x*=0.9 and 0.67) was prepared by varying the Ni:Fe atomic ratio of the starting materials (see Methods for details). The formation of Ni_*x*_Fe_1−*x*_Se_2_ was supported by X-ray photoelectron spectroscopy (XPS). In both Ni 2*p*3/2 and Fe 2*p*3/2 spectra (Fig. 2b and Supplementary Fig. 2), the main peaks have binding energies between those of the corresponding metal and metal oxides^11^,^22^,^23^. For example, the main peak at 853.5 eV in the Ni 2*p*3/2 spectra has a binding energy between nickel metal (852.6 eV) and nickel oxides (853.7–854.9 eV). These binding energies are indicative of metal selenides. The Se 3*d*5/2 binding energy of Ni_*x*_Fe_1−*x*_Se_2_ is 55.2 eV, red-shifted from 55.4 eV for elemental Se (Fig. 2c)^11^. The minor broad peaks in Fig. 2b,c were attributed to surface impurities NiO_*x*_, FeO_*x*_ and SeO_2_. This assignment is consistent with the previous finding that the surface of metal selenides is prone to oxidation by air^11^.

**Figure 3:**
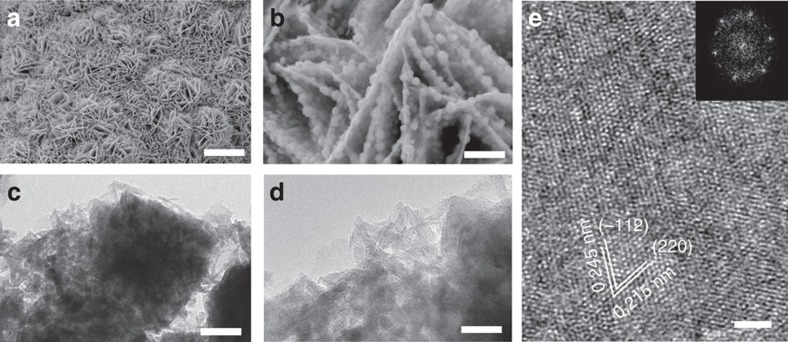
Structural characterization of Ni_*x*_Fe_1−*x*_Se_2_. (**a**) SEM image of Ni_*x*_Fe_1−*x*_Se_2_ nanoplates. (**b**) Magnified SEM image showing the nanoparticles grown on the nanoplates. (**c**) TEM image of a nanoparticle. (**d**) Magnified TEM image showing the nanosheets that make up the nanoparticles. (**e**) HRTEM image of a nanosheet. The inset shows the FFT image of (**e**). Scale bars: (**a**) 10 μm; (**b**) 1 μm; (**c**) 50 nm; (**d**) 20 nm; and (**e**) 2 nm. FFT, fast-Fourier transform.

The morphology of as-prepared Ni_*x*_Fe_1−*x*_Se_2_ (*x*=0.8) was first characterized by scanning electron microscopy (SEM). At the microscale, Ni_*x*_Fe_1−*x*_Se_2_ inherited the morphology of NiFe LDH (Fig. 3a,b and Supplementary Fig. 3), and after selenization, nanoplates of ∼150 nm in thickness were formed. Higher-magnification images, however, revealed that in contrast to NiFe LDH nanoplates which had a smooth surface (Supplementary Fig. 3b), Ni_*x*_Fe_1−*x*_Se_2_ nanoplates were composed of numerous nanoparticles (Fig. 3b). The TEM image further revealed the porous hierarchical morphology of Ni_*x*_Fe_1−*x*_Se_2_ (Fig. 3c). The nanoparticles were made of ultrathin nanosheets (Fig. 3d). HRTEM image indicated the single-crystal nature of each nanosheet (Fig. 3e). Lattice fringes were observed, with inter-planar distances of ∼0.245 and ∼0.215 nm, corresponding to the (−112) and (220) planes, respectively. The basal plane of the nanosheet is (111), which was confirmed by fast-Fourier transform image (inset in Fig. 3e). Elemental mapping analysis (Supplementary Fig. 4) showed that Ni, Fe and Se were homogenously distributed, consistent with the formation of single-phase Ni_*x*_Fe_1−*x*_Se_2_. The atomic ratio of Se/Ni/Fe is ∼7.3:2.6:1.

### *In situ* transformation

**Figure 4:**
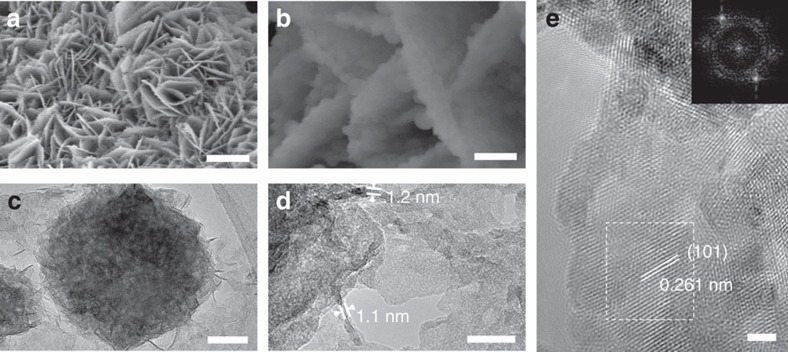
Structural characterization of Ni_*x*_Fe_1−*x*_Se_2_-DO. (**a**) SEM image of Ni_*x*_Fe_1−*x*_Se_2_-DO nanoplates. (**b**) Magnified SEM image showing the nanoparticles grown on nanoplates. (**c**) TEM image of a nanoparticle. (**d**) Magnified TEM image showing the nanosheets that make up the nanoparticles. (**e**) HRTEM image of nanosheets. The inset shows the FFT image of the rectangle region in **e**. Scale bars: (**a**) 10 μm; (**b**) 1 μm; (**c**) 50 nm; (**d**) 10 nm; and (**e**) 2 nm. FFT, fast-Fourier transform

As mentioned above, we expected that Ni_*x*_Fe_1−*x*_Se_2_ would be converted to NiFeO_*x*_ under OER conditions. This Ni_*x*_Fe_1−*x*_Se_2_-derived oxide (Ni_*x*_Fe_1−*x*_Se_2_-DO; we use ‘DO' to designate the selenide-derived oxide from here on) was in fact our target catalyst. Our hope was that Ni_*x*_Fe_1−*x*_Se_2_-DO would inherit the potentially beneficial nanostructure of Ni_*x*_Fe_1−*x*_Se_2_. Thus, Ni_*x*_Fe_1−*x*_Se_2_-DO was obtained by subjecting Ni_*x*_Fe_1−*x*_Se_2_ to galvanostatic scan at the current density of 5 mA cm^−2^ until a stable potential was reached. SEM images (Fig. 4a,b) showed that Ni_*x*_Fe_1−*x*_Se_2_-DO had a similar overall morphology to Ni_*x*_Fe_1−*x*_Se_2._ TEM images (Fig. 4c,d) showed the nanosheets of Ni_*x*_Fe_1−*x*_Se_2_-DO were only about 1–2 nm in thickness. HRTEM image (Fig. 4e) showed lattice fringes of Ni_*x*_Fe_1−*x*_Se_2_-DO, which were different from those of Ni_*x*_Fe_1−*x*_Se_2_. The inter-planar distance of 0.261 nm Ni_*x*_Fe_1−*x*_Se_2_-DO was indexed to the (101) planes of metal hydroxides (for example, LDH or α-Ni(OH)_2_).

**Figure 5:**
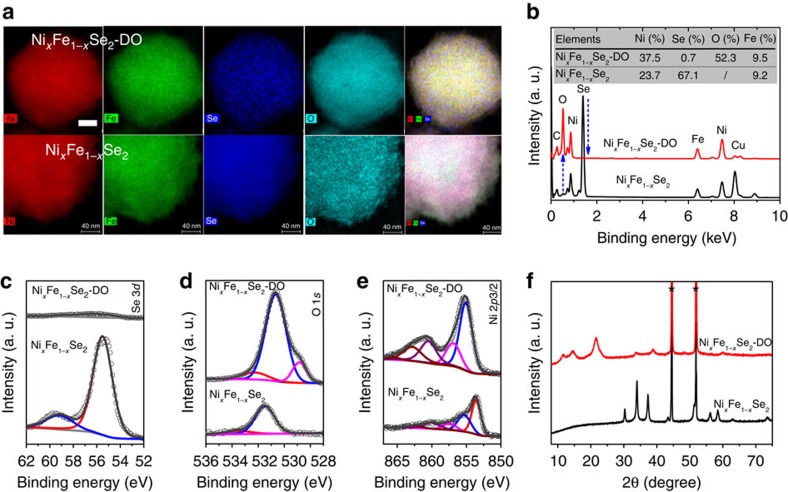
Comparison of Ni_*x*_Fe_1−*x*_Se_2_-DO with Ni_*x*_Fe_1−*x*_Se_2_. (**a**,**b**) Elemental mapping images (**a**) and the corresponding EDS (**b**). Colour in elemental mapping images: red for Ni; green for Fe; blue for Se; cyan for O. Scar bar, 40 nm. The inset in the spectra shows the elemental atomic percentages. (**c**–**e**) High-resolution XPS spectra of Se 3*d* (**c**) O 1*s* (**d**) and Ni 2*p*3/2 (**e**). In O1*s*, the minor peaks of 528.8 and 532.3 eV can be assigned to lattice oxygen (O^2−^) and adsorbed molecular water, respectively. (**f**) PXRD patterns. The asterisks ‘*' mark the diffraction peaks from the nickel foam substrate. EDS, energy-dispersive spectroscopy.

Elemental mapping was conducted to examine the change of composition after the *in situ* transformation. From Ni_*x*_Fe_1−*x*_Se_2_ to Ni_*x*_Fe_1−*x*_Se_2_-DO, Ni and Fe remained homogeneously distributed, Se was removed, while oxygen was incorporated (Fig. 5a). Based on the energy-dispersive X-ray spectra (EDS), the Se content decreased from 67.1% in Ni_*x*_Fe_1−*x*_Se_2_ to 0.7% in Ni_*x*_Fe_1−*x*_Se_2_-DO, and the oxygen content increased from undetectable in Ni_*x*_Fe_1−*x*_Se_2_ to 52.3% in Ni_*x*_Fe_1−*x*_Se_2_-DO (Fig. 5b). The compositional change was further confirmed by high-resolution XPS spectra (Fig. 5c–e). The Se 3*d* peak nearly vanished while the O 1*s* peak increased dramatically in intensity. The main peak in the O 1*s* spectrum was at 530.6 eV, attributed to Ni-OH or Fe-OH (ref. 24). On the basis of the XPS spectra, the atomic percentages of O and Se were ∼47.7% and 1.29% in Ni_*x*_Fe_1−*x*_Se_2_-DO, in agreement with the EDS results. The Ni 2p3/2 peak was shifted from 853.5 eV in Ni_*x*_Fe_1−*x*_Se_2_ to 855.1 eV in Ni_*x*_Fe_1−*x*_Se_2_-DO, also consistent with the formation of NiFeO_*x*_. Finally, the PXRD pattern of Ni_*x*_Fe_1−*x*_Se_2_-DO (Fig. 5f) also indicated the disappearance of Ni_*x*_Fe_1−*x*_Se_2_ and the formation of a crystalline NiFeO_*x*_ phase. Based on previous studies of the electrochemical oxidation of NiSe and MoSe_2_ in basic solutions^25^, the *in situ* transformation is proposed to occur via the following pathways:


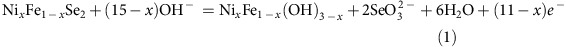



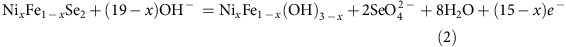


### Oxygen-evolution catalysis

**Figure 6:**
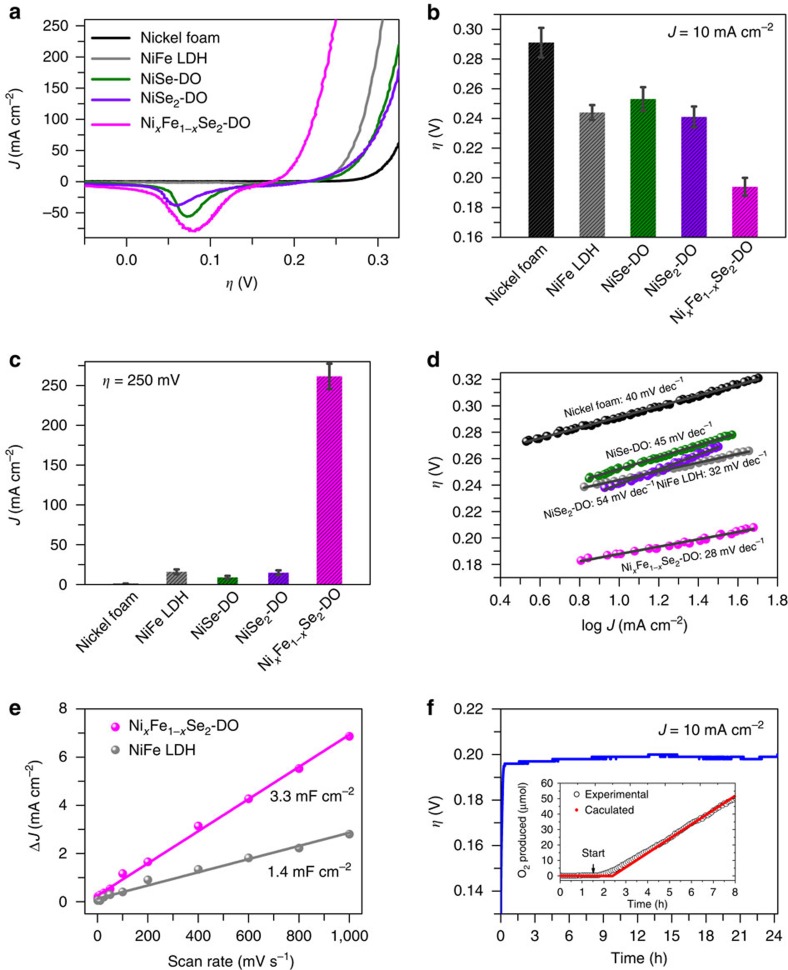
Electrochemical characterization. (**a**) Polarization curves; (**b**) overpotential required for *J*=10 mA cm^−2^; (**c**) current densities at *η*=250 mV; and (**d**) Tafel plots for Ni_*x*_Fe_1−*x*_Se_2_-DO, NiFe LDH, NiSe_2_-DO, NiSe-DO and NF. The error bar represents the range of results from three independent measurements. (**e**) Capacitive *J* versus scan rate for Ni_*x*_Fe_1−*x*_Se_2_-DO and NiFe LDH. The linear slope is equivalent to twice of the double-layer capacitance *C*_dl_. (**f**) Chronopotentiometric measurements of OER at 10 mA cm^−2^ using Ni_*x*_Fe_1−*x*_Se_2_-DO as catalyst. The inset shows the calculated versus actual oxygen production catalyzed by at a constant current of 1 mA. The calculated value represent the expected amount of O_2_ assuming a quantitative Faradaic yield for O_2_ formation.

The electrocatalytic activity of Ni_*x*_Fe_1−*x*_Se_2_-DO, NiFe LDH, NiSe_2_-DO, NiSe-DO and Ni foam (NF) towards OER in 1 M KOH oxidation was measured and compared (see Methods for details). As shown in Fig. 6a, Ni_*x*_Fe_1−*x*_Se_2_-DO is the best catalyst among the five compounds, giving much higher current density (*J*) at the same overpotential (*η*). To reach *J*=10 mA cm^−2^, Ni_*x*_Fe_1−*x*_Se_2_-DO required an overpotential of only 195 mV, which was 49, 46, 58 and 96 mV less than that of NiFe LDH, NiSe_2_-DO, NiSe-DO and NF, respectively (Fig. 6b). The current density at *η*=250 mV was 262 mA cm^−2^, which was 16-, 18-, 29- and 262-fold higher than those of NiFe LDH, NiSe_2_-DO, NiSe-DO and NF, respectively (Fig. 6c). In fact, the activity of Ni_*x*_Fe_1−*x*_Se_2_-DO is superior to other state-of-the-art catalysts (Supplementary Table 1). NiFe LDH has been regarded as the most active OER catalyst in alkaline conditions. Lu *et al*.^26^ reported the overpotential of ∼256 mV for 10 mA cm^−2^ by NiFe LDH grown on Ni foam. The catalytic activity of NiFe LDH was further improved by applying an improved electrosynthesis method^27^. However, an overpotential of 224 mV for 10 mA cm^−2^ was still required. The activity of Ni_*x*_Fe_1−*x*_Se_2_-DO compares favourably even to the best hybrid catalysts made of NiFeO_*x*_ and carbon nanomateirals. Ma *et al*.^28^ reported that the NiFe hydroxide/graphene superlattice reached 10 mA cm^−2^ at an overpotential of 210 mV. Hou *et al*.^29^ reported that exfoliated graphene/Co0.85Se/NiFe LDH composites catalysed OER with an overpotential of 203 mV for 10 mA cm^−2^. The most active hybrid catalyst, r-GO/NiFe LDH, was reported by by Long *et al*.,^30^ which gave 10 mA cm^−2^ at an overpotential of 195 mV. It was proposed that the activity of these hybrid materials originated from a synergetic effect between NiFeO_*x*_ and carbon nanomaterials (CNT, r-GO and carbon quantum dots). Thus, our Ni_*x*_Fe_1−*x*_Se_2_-DO is until now the most active single-phase catalyst, and even higher activity might be achieved by coupling it to carbon nanomaterials. The influence of Ni:Fe atomic ratio on the OER activity was probed. The highest activity was obtained at *x*=0.8, while the samples with *x*=0.9 and 0.67 exhibited only modestly lower activity (Supplementary Fig. 5). This result is consistent with previous reports that that the activity of NiFeO_*x*_ varied only slightly when the Fe:Ni ratio was changed from 0.1 and 0.55 (refs 18, 31).

By plotting overpotential against log (*J*), the kinetic parameters of OER by the five catalysts were calculated (Fig. 6d). The Ni_*x*_Fe_1−*x*_Se_2_-DO has a Tafel slope of about 28 mV dec^−1^, close to that of NiFe LDH (32 mV dec^−1^), but much smaller than the other catalysts (40–54 mV dec^−1^). We further compared the electrochemical surface area (ECSA), estimated from their double-layer capacitance (*C*_dl_) of Ni_*x*_Fe_1−*x*_Se_2_-DO and NiFe LDH. The ECSA of Ni_*x*_Fe_1−*x*_Se_2_-DO is 2.4-fold of the ECSA of NiFe LDH (Fig. 6e) that does not account for the 16-fold higher catalytic activity of the former. We attribute the superior activity of Ni_*x*_Fe_1−*x*_Se_2_-DO to its desirable nanostructure. As shown in Fig. 4, Ni_*x*_Fe_1−*x*_Se_2_-DO nanoparticles are made of ultrathin nanosheets. If the edges of NiFeO_*x*_ are the active sites, then a higher number of active sites is expected on Ni_*x*_Fe_1−*x*_Se_2_-DO than other forms of NiFeO_*x*_. To provide additional support for this hypothesis, the microstructures of Ni_*x*_Fe_1−*x*_Se_2_-DO was compared with NiFe LDH, one of the most active forms of NiFeO_*x*_ (refs 26, 27). SEM images show that NiFe LDH is made of large single-crystalline nanoplates whereas Ni_*x*_Fe_1−*x*_Se_2_-DO is made of highly porous nanoplates consisting of ultrathin nanosheets (Supplementary Fig. 6). Thus, Ni_*x*_Fe_1−*x*_Se_2_-DO's particle size is much smaller than NiFe LDH. The porosity of Ni_*x*_Fe_1−*x*_Se_2_-DO and NiFe LDH were probed by N_2_ adsorption–desorption measurements. Compared with NiFe LDH, more pores were formed in Ni_*x*_Fe_1−*x*_Se_2_-DO (Supplementary Fig. 7). More importantly, an additional sharp peak was observed at a size range of 2.5–4 nm for Ni_*x*_Fe_1−*x*_Se_2_-DO, indicating the formation of tiny nanopores. The nanoporosity resulted in a high Brunauer-Emmett-Teller (BET) surface area of 109 m^2^ g^−1^ for Ni_*x*_Fe_1−*x*_Se_2_-DO, which is 8.3 times higher than that of NiFe LDH. The higher porosity and smaller particle size of Ni_*x*_Fe_1−*x*_Se_2_-DO compared with NiFe LDH would lead to higher number of edge sites in the former, which might explain its superior catalytic activity. The molecular origin of this activity is subject to further studies.

The stability of OER catalysed by the Ni_*x*_Fe_1−*x*_Se_2_-DO electrode was tested at a constant current density *J* of 10 mA cm^−2^ for 24 h. Figure 6f shows that after an activation period of 0.5 h, the overpotential remained at ∼195 mV during 24 h. The Faradaic efficiency of OER was determined using a fluorescence O_2_ probe. A quantitative yield was found quantitative during a 6.5 h electrolysis experiment (inset in Fig. 6f).

## Discussion

In summary, using NiSe as a representative example of metal selenides, we show that metal selenides are converted into metal oxides or hydroxides under OER conditions. These oxides or hydroxides are responsible for the catalytic activity of metal selenides in OER. Taking advantage of this *in situ* transformation, we prepared for the first time a nickel iron diselenide (Ni_*x*_Fe_1−*x*_Se_2_) and used it as a templating precursor to ultrathin nanosheets of the corresponding oxide, Ni_*x*_Fe_1−*x*_Se_2_-DO. A current density of 10 mA cm^−2^ is obtained at an overpotential of only 195 mV in alkaline solutions using Ni_*x*_Fe_1−*x*_Se_2_-DO as the catalyst, making it one of the most active OER catalyst reported to date. The templating approach described here might be applicable for the synthesis of other metal oxide-based nanomaterials.

## Methods

### Materials synthesis

Both NiSe_2_ and Ni_*x*_Fe_1−*x*_Se_2_ were prepared through two steps: (i) metal hydroxide precursors were grown on nickel foam via a hydrothermal method; and (ii) hydroxide precursors were converted into diselenides via a solvothermal selenization treatment. ‘*x*' represents the Ni ratio in the total metal elements of the starting solution. Typically, for Ni_*x*_Fe_1−*x*_Se_2_ (*x*=0.8), Ni(NO_3_)_2_·6H_2_O (2.0 mmol, 582 mg), FeSO_4_·7H_2_O (0.5 mmol, 139 mg), NH_4_F (10 mmol, 370 mg) and urea (25 mmol, 1,501 mg) were dissolved in H_2_O (40 ml). The as-obtained solution together with one piece of nickel foam (5 × 30 mm) were then sealed in a 45 ml Teflon-lined stainless steel autocalve and heated at 120 °C in an electric oven for 16 h. After being washed thoroughly with distilled water and absolute ethanol, the as-prepared nickel foam coated with NiFe hydroxides was submerged into another 45 ml autocalve containing Se (3.75 mmol, 296 mg), NaOH (7.5 mmol, 300 mg), hydrazine (0.14 ml) and dimethylformamide (DMF) (25 ml). After keeping at 180 °C for 1 h, Ni_*x*_Fe_1−*x*_Se_2_ was obtained. To synthesize Ni_*x*_Fe_1−*x*_Se_2_ (*x=*1, 0.9 and 0.67) with variable Ni:Fe ratios, Ni(NO_3_)_2_·6H_2_O and FeSO_4_·7H_2_O with the desired molar ratio were dissolved in water with a total quantity of 2.5 mmol. When *x*=1, NiSe_2_ was obtained.

### Materials characterization

PXRD patterns were recorded on an X'Pert Philips diffractometer with monochromatic Cu_K*α*_ radiation (*λ*=1.540598 Å) and a fast Si-PIN multi-strip detector. As-synthesized NiSe_2_ and Ni_*x*_Fe_1−*x*_Se_2_ were used directly for PXRD measurements. Morphology and microstructure was examined by a Phillips (FEI) XLF-30 FEG SEM and a FEI Tecnai Osiris TEM equipped with high-brightness field emission gun (XFEG). Energy-dispersive X-ray spectroscopy (EDX) mapping images were taken under a scanning TEM modal. Samples for TEM were prepared by drop-drying the samples from their diluted ethanol suspensions onto carbon-coated copper grids. XPS measurements were performed on a PHI5000 VersaProbe II XPS system by Physical Electronics (PHI) with a detection limit of one atomic per cent. Monochromatic X-rays were generated by an Al Kα source (14,867 eV). The diameter of the analysed area is 10 μm. N_2_ adsorption–desorption measurements were conducted on Micromeritics 3Flex adsorption analyser at 77K. Before the measurements, the samples were degassed at 100 °C under vacuum for 1 h. Pore size distributions were calculated by the Barrett-Joyner-Halenda (BJH) method from the desorption branches of the isotherms.

### Electrochemical measurement

Electrochemical characterizations including cyclic voltammetry (CV), LSV and chronopotentiometry were carried out on a Gamry Reference 3000 electrochemical instrument using a three-electrode electrochemical system. A 1 M KOH solution was used as electrolyte, and an Ag/AgCl electrode with saturated KCl filling solution and Pt wire were used as reference and counter electrodes, respectively. For catalyst grown on nickel foam (NiSe_2_, Ni_*x*_Fe_1−*x*_Se_2_ and NiFe-LDH), they were used as work electrodes directly. Hot glue was employed to fix the working area at 0.2 cm^−2^ (0.5 × 0.4 cm). Before test, the reference electrode was measured against another unused Ag/AgCl reference electrode stored in saturated KCl solution. Calibration of Ag/AgCl reference electrodes was done by measuring the reversible hydrogen electrode (RHE) potential using a Pt electrode under a H_2_ atmosphere. During the test, Ag/AgCl reference electrode was constructed to a double-junction electrode to minimize contact between KOH and KCl. CVs were performed at a scan rate of 1 mV s^−1^, and the average of the two potentials at which the current crossed zero was taken to be the thermodynamic potential for the hydrogen electrode reaction, In 1 M KOH electrolytes, *E*_vs.RHE_=*E*_vs.Ag/AgCl_+1.009 V, and overpotential for OER was *η*=*E*_vs.RHE_−1.23 V=*E*_vs.Ag/AgCl_−0.221 V. Ohmic drop correction was performed using the current interrupt method by the potentiostat. Before recording the catalytic activity, catalysts were activated by a chronopotentiometry scan with constant current density of 5–10 mA cm^−2^ until reaching a stable state. Normally, we set the pretreatment time at 12 h. Following the preconditioning, two cycles of LSVs were measured at a scan rate of 1 mV s^−1^. Tafel slopes were calculated based on the LSV curves by plotting overpotential against log(current density). Chronopotentiometry measurements were performed to evaluate the long-term stability. The ECSA was determined by measuring the capacitive current associated with double-layer charging from the scan-rate dependence of CVs. For this, the potential window of CVs was 0.2–0.3 V versus Ag/AgCl. The scan rates were 5, 10, 25, 50, 100, 200, 400, 600, 800 and 1,000 mV s^−1^. The double-layer capacitance (*C*_dl_) was estimated by plotting the Δ*J*=(*J*_a_−*J*_c_) at 0.25 V versus Ag/AgCl against the scan rate. The linear slope is twice of the double-layer capacitance *C*_dl_. The measurements of O_2_ were performed using an Ocean Optics Multifrequency Phase Fluorimeter (MFPF-100) with a FOXY-OR 125 probe (see in Supplementary Methods).

### Data availability

The data that support the findings of this study are available from the corresponding authors upon request.

## Additional information

**How to cite this article:** Xu, X. *et al*. A nickel iron diselenide derived efficient oxygen evolution catalyst. *Nat. Commun.* 7:12324 doi: 10.1038/ncomms12324 (2016).

## Supplementary Material

Supplementary Information Supplementary Figures 1-7, Supplementary Table 1, Supplementary Methods and Supplementary References
