# Supplementary material for: A nickel iron diselenide-derived efficient oxygen-evolution catalyst
Source: Nat Commun. 2016 Aug 9;7:12324. doi: 10.1038/ncomms12324 (PMC4980491; doi:10.1038/ncomms12324)
Supplement: Supplementary Information — Supplementary Figures 1-7, Supplementary Table 1, Supplementary Methods and Supplementary References [file ncomms12324-s1.pdf]

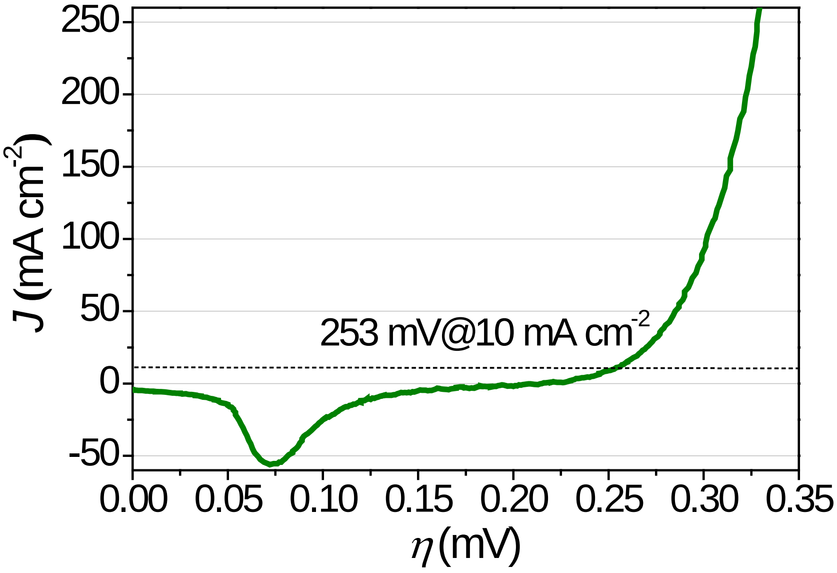

Supplementary Figure 1 | Polarization curve of NiSe.

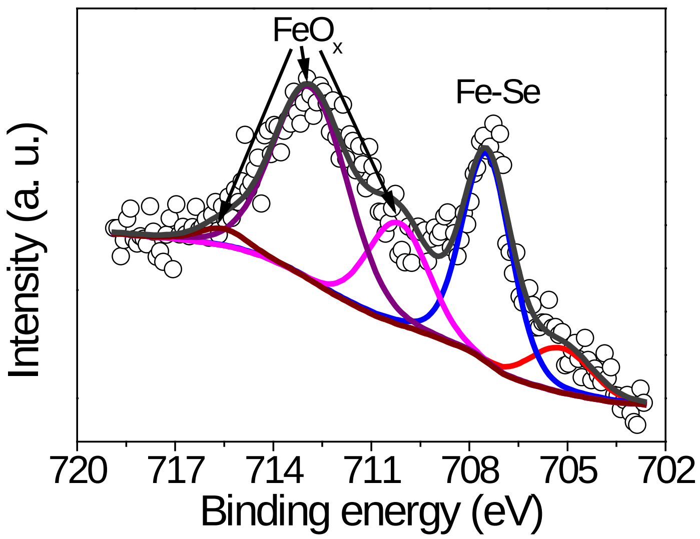

Supplementary Figure 2 | High resolution XPS Fe 2p3/2 spectra for $Ni_xFe_{1-x}Se_2$.

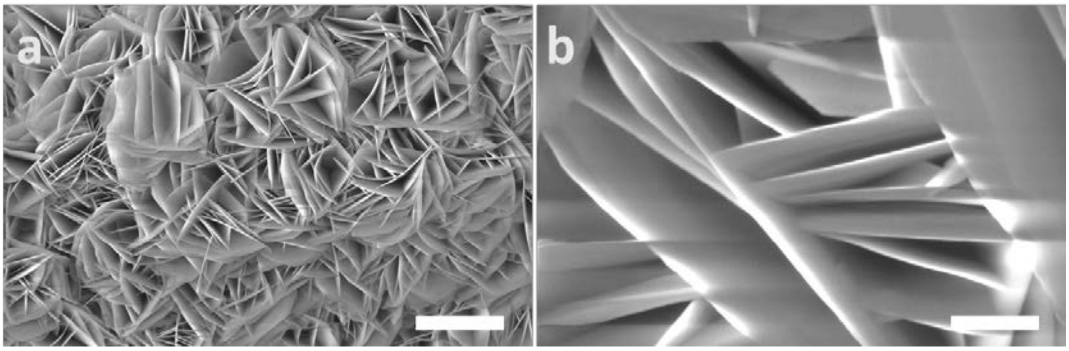

Supplementary Figure 3 | SEM images of NiFe LDH grown on Ni foam. Scale bar: **a**, 10 μm; **b**, 1 μm.

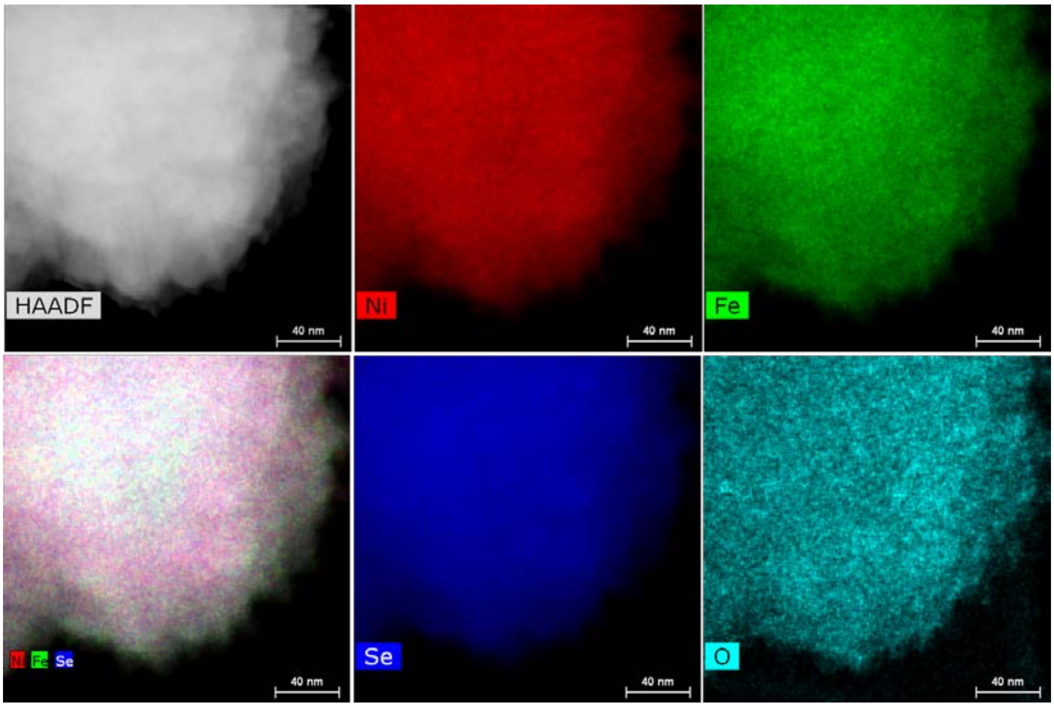

Supplementary Figure 4 | Elemental mapping images of $Ni_xFe_{1-x}Se_2$.

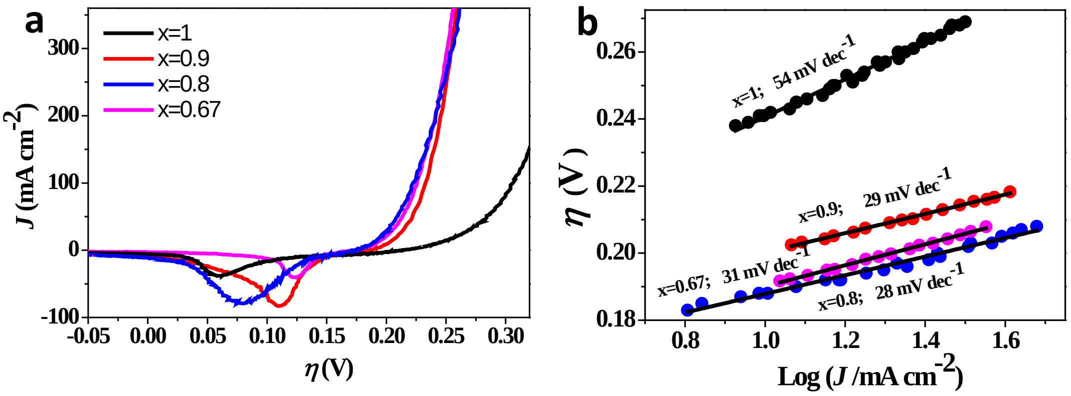

Supplementary Figure 5 | Electrochemical characterizations of $Ni_xFe_{1-x}Se_2$-DO (x=1, 0.9, 0.8, and 0.67). a, Polarization curves; **b**, Tafel plots.

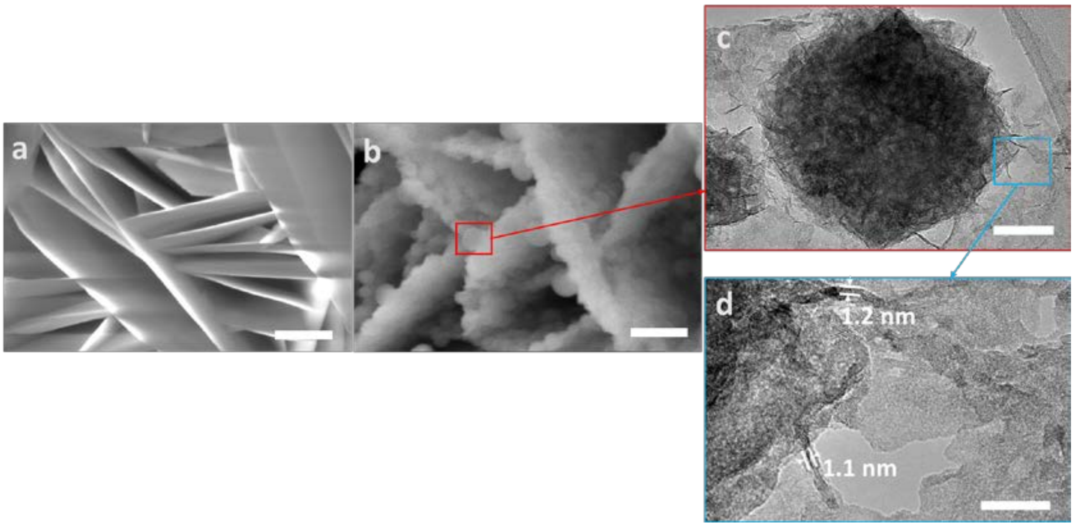

Supplementary Figure 6 | Structural comparison of NiFe LDH and $Ni_xFe_{1-x}Se_2$-DO. **a** and **b**, SEM images of NiFe LDH and $Ni_xFe_{1-x}Se_2$-DO. **c** and **d**, TEM images showing the highly porous nanoplates consisting of ultrathin nanosheets. Scale bars: **a**, 1 μm; **b**, 1 μm; **c**, 50 nm; **d**, 10 nm.

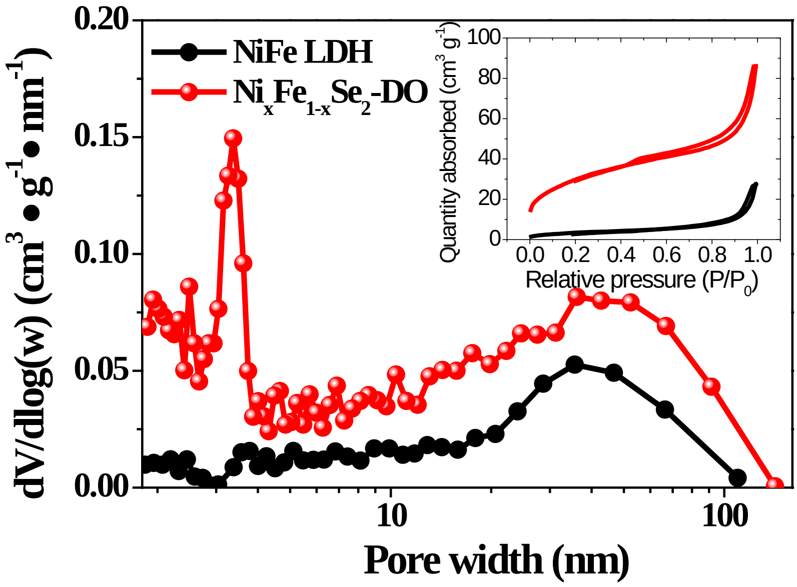

Supplementary Figure 7 | Pore size distribution (BJH model) of NiFe LDH and $Ni_xFe_{1-x}Se_2$-DO. Inset shows the N_2 adsorption-desorption isotherm.

Supplementary Table 1. Comparison of catalytic performance with reported Ni, NiFe based catalysts and IrO_2 nanoparticles.

Materials	Support	Electrolyte	Loading (mg cm^{-2})	$\eta_{@10\ mA\ cm^{-2}}$ (mV)	$J_{@250\ mV}$ (mA cm^{-2})	Tafel solpe	Reff
$Ni_xFe_{1-x}Se_2$-DO	Ni foam	1M KOH	~4.1‡	195	~262	28	This work
$NiSe_2$-DO	Ni foam	1M KOH	~4.1‡	241	15	54	This work
NiFe LDH	Ni foam	1M KOH	~8.3	244	16	32	This work
Ni_3Se_2	Cu foam	1M KOH	3	284	~4	80	[1]
Ni_3Se_2	Ni foam	0.3M KOH	/	~270	~6	99	[2]
NiSe	Ni foam	1M KOH	2.8	~251	7.3	64	[3]
NiFe hydroxides	Ni foam	1M KOH	/	245	15	28	[4]
NiFe LDH	Ni foam	1M KOH	/	240	~10	/	[5]
NiFe LDH	Ni foam	1M KOH	~1	256	7.6	50	[6]
NiFe LDH	Ni foam	1M KOH	1	224	33	53	[7]
NiFe LDH/CNT	CFP†	1M KOH	0.25	~247	12	31	[8]
NiFe LDH/r-GO	Ni foam	1M KOH	0.25/1	200/210	100	40	[9]
NiFe LDH/r-GO	Ni foam	1M KOH	0.25	195	257	39	[10]
EG/$Co_{0.85}$Se/NiFe LDH	Griphite foil	1M KOH	4	203	67	57	[11]
IrO_2	Ni foam	1M KOH	0.7	285	1.7	46	[12]
IrO_2	CFP†	1M KOH	3.3	264	5	47	[13]

† Carbon fiber paper denoted as CFP; ‡ Assume that all the metal elements remained in the final selenides derived oxides.

Supplementary Methods

Materials

Nickel nitrate hexahydrate ($Ni(NO_3)_2{\cdot}6H_2O$, ≥99%, Fluka), iron(II) sulfate heptahydrate ($FeSO_4{\cdot}7H_2O$, ≥99%, Sigma-Aldrich), ammonium fluoride (NH_4F, ≥98%, Sigma-Aldrich), urea ($CO(NH_2)_2$, ≥99%, Acros), selenium powder (Se, ≥99.5%,Acros), sodium hydroxide (NaOH, REACTOLAB SA), hydrazine monohydrate ($N_2H_4{\cdot}H_2O$, ≥64-65 wt %, Sigma-Aldrich) and N, N-dimethylformamide (DMF, C_3H_7NO, ≥99.8%, Sigma-Aldrich) were all used as received without any purification. Nickel foam (purity 95%, porosity 95%, thickness 1.6 mm, bulk density 0.45 $g{\cdot}cm^{-3}$, GoodFellow Cambridge Ltd.) was cleaned ultrasonically for ten minutes with acetone and then with HCl (37 wt%) solution, and subsequently washed with water and ethanol. Deionized water (>18 MΩ·cm resistivity) obtained from a Milli-Q integral water purification system (Merck Millipore Corporation) was used throughout all experiments.

Faradaic Efficiency Test

The measurements of O_2 were performed using an Ocean Optics Multifrequency Phase Fluorimeter (MFPF-100) with a FOXY-OR 125 probe. A linear 2-point calibration curve was created using air (20.9% O_2) and a sealed glass cell that had been purged with N_2 for more than 2 hours (0% O_2). Electrolysis experiments were performed in an airtight H shape cell. The platinum counter electrode was inserted into one side of the cell. The modified working electrode, an Ag/AgCl reference electrode (has a potential of 0.197 V vs. NHE) and a magnetic stirring bar were inserted in the other side. The cell was filled with 1 M KOH solution until the head space of the compartment containing the working electrode is about 7.9 mL. The oxygen probe was inserted into this head space. The cell was purged with nitrogen for 20 min, and the O_2 concentration in the headspace was then monitored for 5 h to establish a baseline. A constant oxidation current density of 14 mA cm^{-2} was passed for 6.5 h. The faradaic yield was calculated from the total amount of charge Q (C) passed through the cell and the total amount of oxygen produced n_{O2}(mol). $Q = t/1000$ (C), where t is the time (s) for the constant oxidation current. The total amount of oxygen produced was measured using the MFPF-100 with a FOXY 125 probe. Assuming that four electrons are needed to produce one O_2 molecule, the Faradaic efficiency can be calculated as follows: Faradaic efficiency = $4F\times n_{O2}/Q = 4000F\times n_{O2}/t$, where F is the Faraday constant.

Supplementary References

1 Shi, J. L., Hu, J. M., Luo, Y. L., Sun, X. P. & Asiri, A. M. Ni3Se2 film as a non-precious metal bifunctional electrocatalyst for efficient water splitting. *Catal Sci Technol* **5**, 4954-4958 (2015).

2 Swesi, A. T., Masud, J. & Nath, M. Nickel selenide as a high-efficiency catalyst for oxygen evolution reaction. *Energy Environ Sci*, **9**, 1771-1782 (2016).

3 Tang, C., Cheng, N., Pu, Z., Xing, W. & Sun, X. NiSe Nanowire Film Supported on Nickel Foam: An Efficient and Stable 3D Bifunctional Electrode for Full Water Splitting. *Angew. Chem.Int. Ed.* **54**, 9351-9355 (2015).

4 Lu, X. Y. & Zhao, C. A. Electrodeposition of hierarchically structured three-dimensional nickel-iron electrodes for efficient oxygen evolution at high current densities. *Nat Commun* **6**, doi: 10.1038/Ncomms7616 (2015).

5 Luo, J. S. *et al.* Water photolysis at 12.3% efficiency via perovskite photovoltaics and Earth-abundant catalysts. *Science* **345**, 1593-1596 (2014).

6 Lu, Z. *et al.* Three-dimensional NiFe layered double hydroxide film for high-efficiency oxygen evolution reaction. *Chem Commun* **50**, 6479-6482 (2014).

7 Li, Z. H. *et al.* Fast electrosynthesis of Fe-containing layered double hydroxide arrays toward highly efficient electrocatalytic oxidation reactions. *Chem Sci* **6**, 6624-6631 (2015).

8 Gong, M. *et al.* An Advanced Ni-Fe Layered Double Hydroxide Electrocatalyst for Water Oxidation. *J. Am. Chem. Soc.* **135**, 8452-8455 (2013).

9 Ma, W. *et al.* A Superlattice of Alternately Stacked Ni-Fe Hydroxide Nanosheets and Graphene for Efficient Splitting of Water. *Acs Nano* **9**, 1977-1984 (2015).

10 Long, X. *et al.* A Strongly Coupled Graphene and FeNi Double Hydroxide Hybrid as an Excellent Electrocatalyst for the Oxygen Evolution Reaction. *Angew. Chem. Int.Ed.* **53**, 7584-7588 (2014).

11 Hou, Y. *et al.* Vertically oriented cobalt selenide/NiFe layered-double-hydroxide nanosheets supported on exfoliated graphene foil: an efficient 3D electrode for overall water splitting. *Energy Environ Sci* **9**, 478-483 (2016).

12 Yan, X. D. *et al.* From Water Oxidation to Reduction: Transformation from NixCo3-xO4 Nanowires to NiCo/NiCoOx Heterostructures. *ACS Appl. Mater. Interfaces* **8**, 3208-3214 (2016).

13 Wang, P., Song, F., Amal, R., Ng, Y. H. & Hu, X. Efficient Water Splitting Catalyzed by Cobalt Phosphide-Based Nanoneedle Arrays Supported on Carbon Cloth. *ChemSusChem* **9**, 472-477 (2016).
